# Feasibility, acceptability, and effects of a web-delivered behavioral parent training intervention for rural parents of children with autism spectrum disorder: A protocol

**DOI:** 10.1371/journal.pone.0307273

**Published:** 2024-08-27

**Authors:** Samantha Ault, Kayla Herbell, Nathan Helsabeck, Kevin Stephenson, Susan M. Breitenstein, Laureen M. Smith

**Affiliations:** 1 College of Nursing, The Ohio State University, Columbus, Ohio, United States of America; 2 Nationwide Children’s Hospital, Columbus, Ohio, United States of America; University of Illinois Medical Center at Chicago: University of Illinois Hospital, UNITED STATES OF AMERICA

## Abstract

Caregivers of children with autism spectrum disorder (ASD) often report higher levels of stress and mental health issues. Support services and parent training programs may help buffer the effects of caring for a child with ASD. However, due to the national lack of trained ASD providers and disparity of ASD support resources available in rural areas, caregivers often go without support. A possible solution to reach caregivers in rural areas is web-based interventions. This paper describes an ongoing pilot study examining the feasibility, acceptability, and preliminary effects on caregiver well-being and disruptive child behaviors for a web-based parent training program (Attend Behavior) for caregivers of young children (ages 2–11 years old) with autism spectrum disorder (ASD) living in rural areas (trial registration NCT05554198). The intervention is available on the internet as well as a downloadable app for mobile phones. Participants will be invited to use the intervention program for 12-weeks. Prior to using the program, participants will be asked to take a baseline survey assessing depressive symptoms (PROMIS Depression Short Form-6a), caregiver stress (Parenting Stress Index-Short Form), child disruptive behaviors (Home Situations Questionnaire-ASD and Aberrant Behavior Checklist). After 12-weeks, participants will be asked to complete a post-intervention survey with the same measurement scales plus questions regarding intervention acceptability, appropriateness, and feasibility (Acceptability of Intervention, Intervention Appropriateness Measure, and the Feasibility of Intervention Measure). Participants are also invited to partake in a brief 1:1 interview with a study team member to give further feedback regarding the intervention. Study retention and participant app usage data will be examined. Information generated from this pilot study will be used to inform a future larger scale randomized control trial of Attend Behavior.

## Introduction

Children with autism spectrum disorder (ASD) have distinct differences in language use, social interaction, and restricted or repetitive behavior, interests, or activities [1]. Parenting a child with ASD can leave parents feeling stressed and fatigued from disruptive behaviors, dependency of the child, and disrupted sleep cycles [2–4]. Parents of children with ASD consistently report higher levels of stress, depression, anxiety, social isolation, and poorer levels of physical health compared to parents of neurotypical children and parents of children with other various chronic health conditions (e.g. cerebral palsy, down syndrome) [2–14]. The severity of ASD and disruptive behaviors are correlated with higher parental stress [3, 15, 16] with formal and informal emotional support sources shown to buffer stress for parents of children with ASD [14, 17–20]. Traditional behavioral treatment approaches, such as applied behavioral analysis (ABA), benefit children with ASD [21]; however, there is limited information about approaches like ABA alleviating parent distress. Treatment approaches are delivered in various formats including parent-mediated delivery seen in parent training (PT) programs [22]. PT programs aim to provide parents with specific skills targeting disruptive child behaviors [23]. Evidence shows that PT is effective in reducing child problem behaviors and parenting stress and depression [24–28]. In addition, there are economic benefits of PT interventions for children with behavioral issues as evidence by significant positive returns for investing in PT for youth at risk or already experiencing behavioral difficulties [29].

### Treatment disparities

Despite evidence of effective ASD interventions for both child and parent outcomes, rural areas lack local support resources and specialized care for children with ASD [30–33]. These disparities include reduced access to services, use of services, and satisfaction and perceived effectiveness of services for children with ASD [32, 34]. The lack of providers in rural areas have resulted in many rural children with ASD relying on the school systems for support despite there being a lack of trained specialists and funding for special education programs in rural schools [35, 36]. This is problematic as it results in children not receiving early intervention prior to entering the school system and parents being fearful for what will happen once they age out of school [37].

Interventions delivered via telehealth services are a response to the lack of service availability. Research evaluating the use of telehealth for those with ASD to provide behavioral therapies and PT programs has increased more recently in response to the COVID-19 pandemic [38]. In a recent review of ASD services including assessments and intervention delivered via telehealth by Ellison et al. [38], eleven studies were noted to use telehealth platforms to deliver PT programs to parents of children with ASD, including one study examining the outcomes of the Research Units in Behavioral Intervention (RUBI) program delivered via telehealth [22]. Overall, results were very promising showing telehealth as an effective route of delivery for interventions [38]. It is important to note that in all eleven studies examining PT programs via telehealth in the review used video conferencing platforms (e.g. Zoom) with a trained intervention provider being present [38]. Cantor et al. [30] noted that less than half of the mental health clinics in the United States (US) provided behavioral health care for children with ASD and only 12.7% reported having a clinician with specialized training for treating children with ASD. With the lack of trained ASD providers noted across the US, telehealth services may expand the reach of services, but overall effect of telehealth remains severely limited with the amount of ASD providers available.

### Self-administered PT

In response to the treatment disparities and the lack of treatment providers nationally, self-administered PT programs may be key to closing the gap in care. PT programs for parents of children with ASD have the potential to be self-administered, meaning parents can access and work through the intervention independently, at their own pace, and on their own preferred time. Such self-administered PT programs may be useful for parents to access independently as a primary intervention when a child is unable to be connected to a specialized provider, on a wait list for a diagnostic assessment or intervention services, or as an adjunct intervention. There remains a lack of research examining the feasibility, accessibility, and efficacy of self-delivered remote PT programs with rural parents. Parsons et al. [39] reviewed parent-mediated interventions delivered remotely for parents of children with ASD living outside urban areas and found seven studies examining interventions that included self-guided material (e.g. DVDs, websites, written material, videos). Some studies included clinician interaction (remote coaching sessions, video conferencing). Findings from the review propose that remotely delivered PT interventions can improve child social behavior and communication skills and increase parental knowledge and skills [39]. However, the overall quality of evidence was low due to various limitations of the studies (e.g. small samples, lack of standardized outcome measures) and only provides preliminary evidence [39].

### Intervention

Attend Behavior is self-administered mobile or web-based PT intervention that educates and encourages the use of evidence-based parenting skills for children with multiple behavior problems such as hyperactivity, impulsivity, mild aggression, and emotional dysregulation. Attend Behavior includes twelve modules that encompass parenting strategies to reduce disruptive child behaviors and increase independence in daily activities. Parents take an assessment that allows Attend Behavior to tailor the intervention material to the individual parent’s needs. There are several parent engagement tools such as automatic and customizable push notifications to remind the parent to engage and apply parenting skills in their everyday life, integrated messaging features for clinicians (when applicable), behavior tracking tools, and graphs to visualize progress. Attend Behavior also includes parent education learning modules with written and video content to learn behavior principles. Therefore, Attend Behavior can be used by the parent independently and other health care providers such as nurses, case managers, and primary care providers have the ability to use this intervention with parents of children with ASD without prior expertise in ASD treatment.

The purpose of Attend Behavior is to provide parents with strategies and support to promote positive parent-child interactions, rather than forcing children to directly conform to neurotypical norms. Attend Behavior teaches evidence-based parenting skills to address behaviors that could present as a safety concern or interfere with important child activities (e.g., personal hygiene, getting dressed, transitioning from one activity to another, leaving the house, attending medical appointments) as well as promote child independence and age-appropriate adaptive skills. The goal is to empower parents and provide support that will work for their own unique and individualized needs. As parents work through Attend Behavior, they can identify individualized goals and application of the behavioral principles rather than having predetermined goals. Table 1 outlines the course topics, learning objectives, and activities for each topic. The Attend Behavior development team worked with experts in the field as well as parents to gain feedback for intervention development.

**Table 1:** Course list and sequence of attend behavior.

Course	Learning Objectives	Activities
ABCs of Behavior	ABC Model Function of Behavior Role of Data Collection	Select Target Behaviors Check-in Assessment Incident Tracking
Prevention Strategies	Antecedent Management Strategy Review	Select Prevention Strategies
Creating Schedules & Routines	Establishing Routines Using Visual Schedules	Configure Schedule Generate Visual Schedule
Using Reinforcers	Intro to Reinforcers Types of Reinforcers Using Reinforcers	Selecting Reinforcers Configuring Reinforcers
Power of Social Reinforcers	Catch Them Doing Good Play Time	Establish Practice Sessions
Using a Token Economy System (TES)	Intro to TES Establishing a TES	Review Token System Tool
Planned Ignoring	Intro to Planned Ignoring Implementation Steps Troubleshooting	Establish Practice Sessions
Improving Compliance with Commands	Non-Compliance Overview Promoting Compliance	Establish Practice Sessions
Replacing Misbehaviors with Functional Communication	Introduction to Functional Communication Training (FCT) Implementing FCT at home	Create a FCT Plan
Introduction to Teaching Skills	Promoting Successful Skill Development Key Strategies for Teaching New Skills	Create a Skill Teaching Plan
Using Prompts to Support New Learning	Introduction to Prompts Steps for Using Successfully Using Prompts	Create Skill Development Data Collection Plan
Effective Use of Time Out	Using Time Out Essential Elements of a Plan	Create Time Out Plan

### Evidence supporting attend behavior

With the sharp increase in smartphone ownership in the last decade, including nearly 80% of adults living in the rural US reporting owning a smartphone, [40] mobile-delivery of PT through smartphone app may be convenient for parents and offer more features than typical Web-based content (e.g. real-time behavior tracking). Attend Behavior is a web-based PT program. Attend Behavior content is based on the RUBI PT Program for reducing disruptive behaviors, henceforth referred to as RUBI [41]. RUBI uses evidence-based parent-focused instruction with the basic principles of ABA. The goal of RUBI is to reduce challenging behaviors and increase adaptive skills through teaching parents behavior management and teaching skills. RUBI has been shown to be feasible and have high acceptability from parents of children with ASD, improve parental knowledge, decrease parental stress, increase child’s daily living skills, and decrease problem behaviors [22, 24, 25, 27, 42]. Historically, RUBI has been conducted in-person at a clinic with a provider and parent. Recently, with the COVID-19 pandemic, many providers had to pivot to use technology to provide services from a distance. In response to this, Shanok et al. [27] completed a feasibility pilot study using synchronous telehealth services for delivering RUBI to parents of children with ASD. The findings of this study showed that the feasibility of RUBI delivered via telehealth for reducing problem behaviors of children with ASD was consistent with prior RUBI studies [27].

Attend Behavior is available to download as a mobile application on the Apple App Store and Google Play. In addition, Attend Behavior is accessible through a web-browser. Attend Behavior is available in both English and Spanish. Providing parents of children with ASD with a self-administered, mobile- or web-based intervention may decrease parental stress by removing barriers of treatment wait times, transportation, childcare, time involvement, and geographical distance that parents of children with ASD living in rural areas face when participating in other in-person PT programs. The Attend Behavior PT intervention aims to reduce a wide variety of child problem behaviors such as mild aggression, defiance, anger outbursts, impulsivity, and hyperactivity. Attend Behavior also includes content on teaching children independent living skills, increasing positive behaviors. Despite being based on the evidence-supported RUBI PT program, there are no current studies published examining the effects of Attend Behavior for parents of children with ASD. There is an overall lack of published studies examining the effects of solely using a mobile-delivered PT program aimed at decreasing disruptive behaviors for children with ASD. Attend Behavior is a paid service, with a monthly subscription cost for parents. Alternatively, agencies or clinicians can purchase Attend Behavior subscriptions for use with parents of their clients, which poses multiple benefits including being able to improve communication with parents, being able to see target behaviors tracked in real time, and in some cases the ability to bill insurance for the use of the therapeutic program.

### Purpose

The primary aim of this study is to test the acceptability and feasibility, of Attend Behavior in a rural population of parents (n = 40) of children (aged 2–11 years old) with ASD. The secondary aim of this study is to test preliminary effects of Attend Behavior on child behaviors and parental mental health in a rural population of parents of children with ASD. Attend Behavior was chosen for testing based on a rigorous review of the existing parenting programs conducted by the study investigator. Parents will provide feedback on the feasibility and acceptability of Attend Behavior and provide feedback on additional elements that they need in an intervention to support them. Preliminary results of this pilot study will influence future larger randomized control trials.

The research questions are:

1. Is Attend Behavior a feasible and acceptable intervention for rural parents/guardians of children with ASD?
2. To what extent does Attend Behavior effect parental mental health (depression and stress) and child outcomes (disruptive behaviors and noncompliance in the home)?

## Materials and methods

### Design

This pilot study is a single group pre/post-intervention design. A mixed-methods approach will be employed with both quantitative and qualitative data analyzed. Quantitative data are collected via surveys using REDCap (Research Electronic Data Capture) [43]. Qualitative data are collected via brief 1:1 semi-structured interviews.

### Participants

We aim to enroll 40 participants. Due to this study’s pilot nature, we will not rely on statistical power for analysis. A sample of 40 participants will likely be met the guideline of 12 or more participants for pilot studies using continuous variables even accounting for potentially high participant attrition [44].

**Fig 1:**
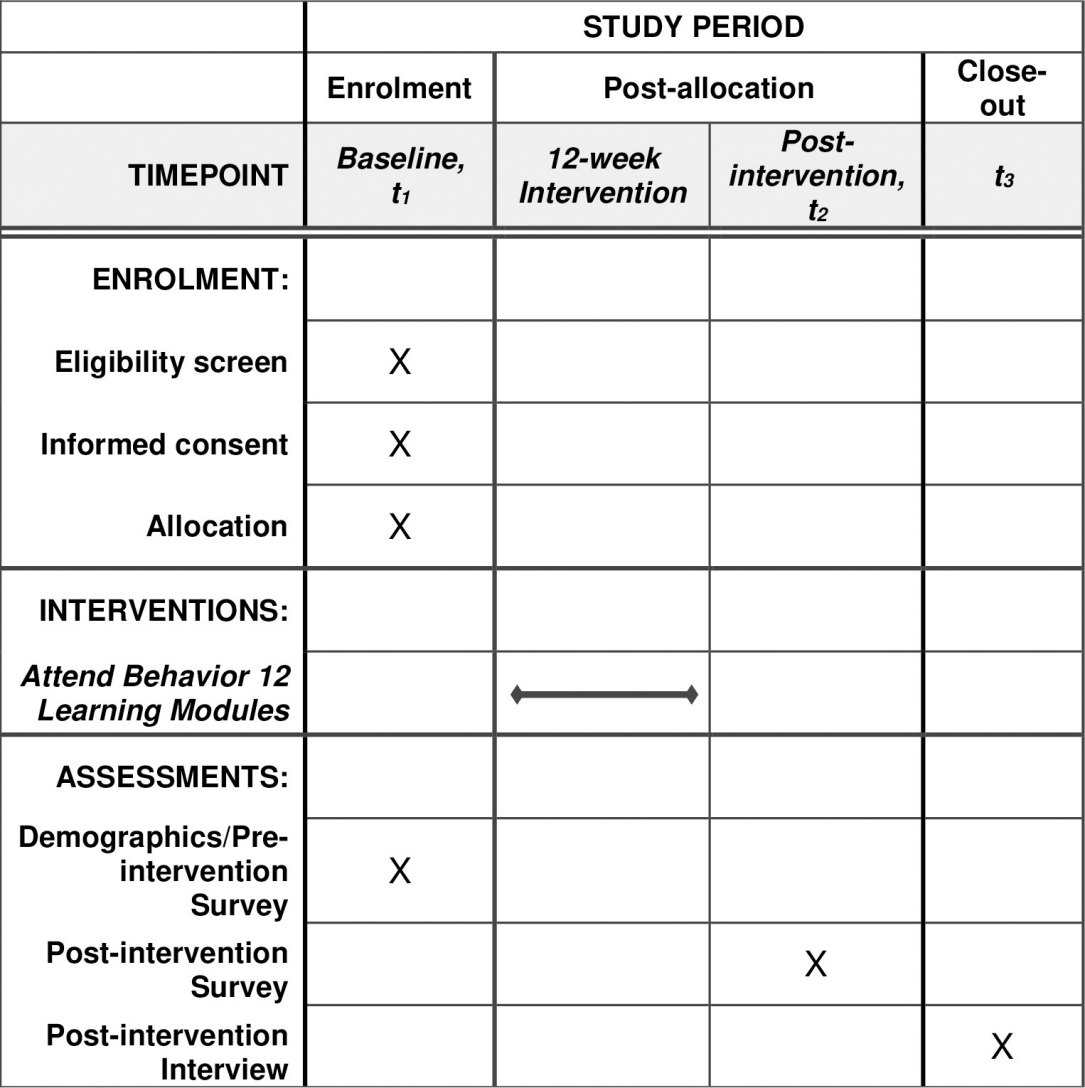
SPIRIT schedule of enrollment, interventions, and assessments.

**Fig 2:**
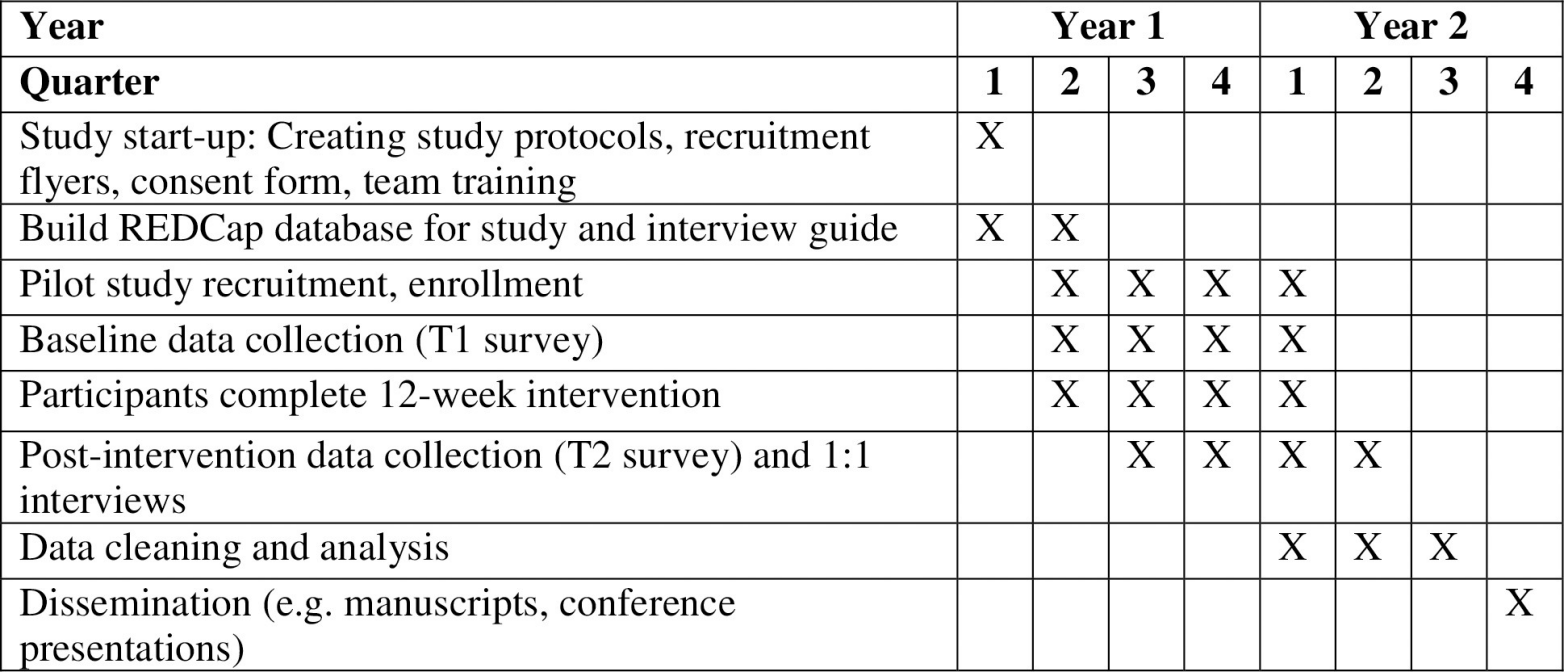
Schedule of enrollment, interventions, and assessments.

Participants will be recruited with the aid from various agencies and organizations with advertisement of the study within the Untied States. Study team members will reach out to rural community-based mental health agencies, schools, libraries, Boards of Developmental Disabilities, and other agencies that are likely to have close contact with families of children with ASD. If the agency or organization is interested, they will be given flyers to disperse to families as they see fit and/or hang in their building(s). In addition, we will partner with The Ohio State University Extension Offices in rural counties to advertise the study. Finally, Facebook will be used for recruitment by both Facebook ads and sending study information to Facebook groups that are specific to parents of children with ASD. Recruitment to the study opened in January 15, 2023 and is anticipated to close by April 2024. See Fig 1 for the SPIRIT schedule of enrollment, interventions, and assessments and Fig 2 for the study timeline.

All interested potential participants will be directed by a link and/or QR code on the recruitment flyers to an eligibility screening questionnaire. Participant inclusion criteria is as follows: The legal guardian of one or more children ages 2–11 years old with a formal diagnosis of ASD by parent report; Child with ASD resides at home with the parent; Parent age is 18 years or older; Reside in a rural (micropolitan or noncore) county; Has access to either a smartphone with the Apple App store or Google Play store or a computer with Internet access; Ability to read in English or Spanish. Participants are excluded if they are currently participating in another behavior PT program or in the past 6-months have been enrolled in another behavior PT program as this could confound the results.

### Outcome measurements

Data in this study will be both quantitative and qualitative in nature. Parents will complete self-reported surveys at baseline (T1) and post-intervention (T2) using REDCap. There will be two baseline surveys, one for parents of children ages 2–5 years old and another for parents of children ages 6–11 years old. Surveys will be available in both English and Spanish. Participants will be emailed links to complete T1 and T2 surveys.

### Demographics

The demographic survey will include both parent and child information that is self-reported by the parent. The Autism Spectrum Rating Scale–Short Form (ASRS-SF) (2–5 year old and 6–18 year old) are both 15-item measurement scales to assess the severity of common behaviors associated with Autism Spectrum Disorder [45]. If the parent has a child that is 2–5 years old, they will be given the survey that includes the ASRS-SF (2–5 year old) form. If the parent has a child that is 6–11 years old, they will be given the survey that includes the ASRS-SF (6–18 year old) form. The ASRS-SF asks parents to rate how often during the past four weeks their child had a specified behavior. The parent is prompted to answer on a Likert scale of 0 = *Never* to 4 = *Very Frequently*. Raw scores can range from 0–60 with higher scores indicating higher rates of behaviors. Based upon the scores, children will be classified into categories of low, average, slightly elevated, elevated, and very elevated. The ASRS-SF (2–5 Years and 6–18 Years) has shown high levels of internal consistency and high discriminative validity when predicting group members of ASD vs. general population [45].

### The aberrant behavior checklist (ABC)

The ABC measures child problem behavior and consists of 58 questions with five subscales: irritability, withdrawal, stereotypic behavior, hyperactivity, and inappropriate speech [46]. Parents are asked to rate how big of a problem specified behaviors have been for their child within the last four weeks on a Likert scale of 0 = *not a at all problem* to 3 = *the problem is severe in degree*. Higher scores indicate higher severity of problem behaviors. The ABC has shown adequate convergent and divergent validity for children with ASD [47]. The ABC has also been validated in Spanish for children with ASD [48]. For this study, only the irritability subscale (ABC-I) which assesses severity of behaviors such as tantrums, self-injurious behaviors, and aggression will be used for analyses. The ABC-I has been used as an outcome measurement in multiple clinicals involving children with ASD, including previous studies examining the effectiveness of RUBI [24, 25, 49, 50].

### The home situations questionnaire–autism spectrum disorder (HSQ-ASD)

The HSQ-ASD has been shown to be a valid and reliable measure of child noncompliance in the home and consists of a 24-item rating (with two subscales: Socially Inflexible and Demand Specific) [51]. For the HSQ-ASD, parents report yes/no if the child has been noncompliant in the past 4 weeks to a variety of situations in the home setting (e.g. “when told to brush teeth”). If the child reports the child was noncompliant in a situation within the last 4 weeks, they are prompted to rate the severity of that problem on a 1–9 Likert scale with higher scores indicating higher severity.

### The PROMIS depression–short form (SF) 6A

The PROMIS Depression- SF 6a is 6-item PROMIS short form measurement that prompts participants to rate how often they have felt emotions associated with depression within the past 7 days on a scale of 1 = *Never* to 5 = *Always*. Higher scores are associated with higher severity of depressive symptoms. The PROMIS Depression forms have shown strong convergent validity with two common and highly validated depression scales, the Patient Health Questionnaire 9-item depression scale (PHQ-9) and Center for Epidemiological Studies Depression scale (CESD) [52–54].

### The parenting stress index–short form (PSI-SF)

The PSI-SF is a 36-item measurement to measure parental stress with three subscales: parental distress (PD), parent-child dysfunctional interaction (PCDI), and difficult child (DC) to yield a total stress score [55]. Higher scores on the PSI-SF indicate higher levels of parental stress. The PSI-SF has shown strong test-rest reliability and internal consistency in high-risk mothers including Spanish speaking mothers [56]. In a study by Zaidman-Zait et al. [57], the PD subscale was shown to work best for parents of children with ASD [57]. Reliability for each PSI-SF subscale varied for parents of children with ASD (PD Cronbach’s α = .88; PCDI Cronbach’s α = .80; DC Cronbach’s α = .82) [57].

### The acceptability of intervention (AIM), intervention appropriateness measure (IAM), and the feasibility of intervention measure (FIM)

The AIM, IAM, and FIM have a total of 12-items answered on a Likert scale of 1 = *completely disagree* to 5 = *completely agree*. The AIM, IAM, and FIM is a brief and reliable measurement to examine implementation success [58, 59]. Higher overall scores correlate with higher rates of participant perceptions of intervention acceptability, appropriateness, and feasibility. Since scores of 4 and 5 indicate that participant agrees or completely agrees, scores of ≥16 for each scale will be used as benchmarks for acceptable, appropriate, and feasible outcomes. Although there is no set cut-off score available for the AIM, IAM, and FIM, the score of ≥16 is chosen because item scores of 4 and 5 suggest that the respondent agrees or completely agrees that Attend Behavior is acceptable, appropriate, or feasible.

The demographic survey and ASRS-SF will only be given at T1. The ABC, HSQ-ASD, PROMIS Depression 6a, and the PSI-SF will be delivered at both T1 and T2. The AIM, IAM, and FIM will only be delivered at T2.

After the T2 survey is complete, participants will be invited to complete an approximately 20-minute exit interview with the PI. The exit interview will be conducted over the phone or zoom according to participant preference. The purpose of the post-intervention interview is to gauge the preliminary feasibility and acceptability of Attend Behavior. The interview will be guided by a semi-structured interview guide. Interviews will be audio-recorded and transcribed. Examples of questions that will be asked during the 1:1 exit interviews include “What did you learn from the program,” “What did you like least about the program,” and “What is the online parenting program missing that could help parents like you who have a child with autism in rural areas?”

### Procedure

The study was approved by The Ohio State University’s Social and Behavioral Institutional Review Board (2021B0434) and the study procedures is registered on clinicaltrials.gov (trial number NCT05554198). Any changes made in the study protocol will be submitted to The Ohio State University’s Institutional Review Board for approval and updated on clinicaltrials.gov.

Once participants have been screened and are eligible, a study team member will contact the participant for an enrollment meeting. The enrollment meeting will take approximately 15 minutes to complete and take place over the phone. The enrollment meeting is comprised of the study team member educating the participant on the study, obtaining verbal consent, verifying participant contact information, and providing an overview of the Attend Behavior intervention. The participant will be emailed a link to the baseline survey after the phone call is complete. In addition to the email with the baseline (T1) survey link, the participant will be emailed a welcome email with a link to the Attend Behavior website which houses a short video created by Attend Behavior explaining the features of the program and how to use the program and a bullet pointed overview of the study. The welcome email will contain copies of the consent form and a contact card for the study team. Once the T1 survey is completed, the participant will be emailed their access code to create their Attend Behavior account. This access code allows participants to use Attend Behavior for free for up to 1 full year.

The enrollment meeting will also include assessing the participant’s preference for email, text messages, and/or Attend Behavior messaging reminders to complete the T2 survey and exit interview. Participants will be offered to schedule the exit interview during the enrollment appointment or an option to schedule the interview at a later time. Participants will be reminded that they can Zoom into the 1:1 interview or complete the interview via phone. Participants will be informed that they will receive a survey link after completing the intervention to complete approximately 12-weeks after initiating the intervention. They will be informed that the Attend Behavior mobile application will be sending them reminders to complete learning modules weekly and to practice skills taught in the modules.

## Data collection and storage

### Quantitative data

Participant including the demographic questionnaire, baseline survey (T1), and the post-intervention survey (T2) will be collected using REDCap (Research Electronic Data Capture) and stored on a secured server [43]. Data from participants’ usage of the Attend Behavior app, will be collected and stored on Attend Behavior’s servers. Encryption used by REDCap to protect respondents includes hashed passwords and SSL certificates between client and server using the secure HTTPS protocol. Data will be downloaded from both servers for cleaning, merging, and analysis using R [60]. There will be no hard copies of data in this study. All data (e.g., audio recordings, surveys) will be housed in the Ohio State University College of Nursing’s R-drive. The R-drive runs Windows Server 2012R2 and is only used to host research files. Electronic access to the research server is restricted to the college LAN or VPN. Only the study team members approved by the Ohio State University Institutional Review board will have access to the project and data on REDCap and the R-drive. Due to the small scale, short duration, and low risks of this study, a data monitoring committee was not deemed to be needed.

We will conduct exploratory data analyses to check data accuracy, examine variable distributions, and summarize sample characteristics. Data anomalies once identified will be fully investigated and remedial strategies will be considered as appropriate. Continuous data that are not normally distributed will be examined for potential outliers and data transformation will be conducted if necessary. Missing data will be examined to determine which mechanism of missingness is most likely, and if appropriate, multiple imputations will be conducted.

### Quantitative analysis plan

To address research question one, *is Attend Behavior a feasible and acceptable intervention for rural parents/guardians of children with ASD*, we will use descriptive statistics of the participant responses to the AIM, IAM, and FIM taken post intervention (T2). Specifically, we will use the mean score across all participants on the AIM to examine acceptability and the mean score across all participants on the FIM to assess feasibility of the intervention. Further, we will report the percent of individuals scoring at or above the benchmark score of 16. Additionally, we will examine the appropriateness of the intervention using the scores from the IAM in a similar manner as the AIM and FIM. Finally, we will report rates of completion of intervention, recruitment, and retention rates. To assess retention, user data from the program will be extracted to examine module completion and program engagement. This will be an exploratory measure as there is limited research on digital engagement for self-administered PT programs for parents of children with ASD. A review of studies examining parent engagement in interventions for underrepresented parents of children with social, emotional, and behavioral disorders showed an average attrition rate of 26% [61]. There was less parent attrition in intervention groups when the interventions were community/home-based, [61] therefore the goal for retention rate is 75%.

To address research question 2, *to what extent does Attend Behavior effect parental mental health (depression and stress) and child outcomes (disruptive behaviors and noncompliance in the home)*, we will summarize pre and post intervention scores using descriptive statistics to and assess change using linear regression. Specifically, we will report descriptive statistics (mean, standard deviation, median, and inn quartile range) for each outcome variable (i.e. ABC-I, HSQ-ASD, PROMIS, and PSI-SF) at preintervention (T1) and post-intervention (T2).

To assess change in outcomes will examine data from all participants who initiated the Attend Behavior modules regardless of how many they complete. To determine if those participants hand a significant change in any of the outcome measures (ABC-I, HSQ-ASD, PROMIS, and PSI-SF), we will employ a residualized change approach where each participants’ outcome measure taken at T2 is regressed on the participants’ baseline (T1) score on the same measure. These regressions will be unadjusted, meaning we will include no covariates in these analyses, as our primary interest is to determine if any intervention effect is present in the data. We will report mean differences, standard errors, 95% confidence intervals, and effect sizes for baseline (T1) to post-intervention (T2) difference. Supplementary analysis will include reexamining these models controlling for number of modules completed to determine if a certain number of models is associated with a larger effect size difference at outcome.

Of note, although the full ABC measurement will be included in the T1 and T2 surveys, only the ABC-Irritability subscale (ABC-I) will be used for assessing outcomes analyses as the intervention does not target the other symptoms included in the other ABC subscales. Other ABC subscales may be used supplementary exploratory analysis, but are not the primary focus of this study.

### Qualitative data and analysis

Qualitative data sources in this study include the exit interview completed at the end of the 12-week intervention. Participants and a study team member will complete the 1:1 semi-guided interview via Zoom or phone. Interviews will take approximately 20–30 minutes. Participants will be given the interview guide questions in advance. Interviews will be audio recorded. All audio files will be transcribed verbatim promptly after the interviews. A member of the study team will review the transcripts of the audio interviews for accuracy. Prior to data analysis, all qualitative sources will be de-identified with identifiers being redacted. All qualitative data will be analyzed using NVIVO.

NVIVO will assist with the organization and coding of the unstructured narrative data. NVIVO has several capabilities including classifying and arranging data and organizing the data into similar concepts to allows for relationships to emerge. The PI and a member of the research team will read all participant interviews. Codes and coding categories will be compared and discrepancies discussed before establishing a codebook.

Due to the qualitative methodology chosen, data analysis will occur simultaneously with data collection. Data saturation will be attained when no new additional data are revealed in the conceptual categories. The data analysis strategy that will be used in the proposed study is content analysis due to the inductive nature of the work. The goal of the content analysis is to offer a detailed depiction of the phenomena of interest using the participant’s own words. The researcher avoids making inferences and instead stays closely aligned with the data by frequently using quotes, nothing themes or codes, and the frequency of the codes. To ensure credibility of the data, the PI will keep a “reflective commentary” log to acknowledge biases and note initial impressions as well as themes as they emerge in the data.

## Monitoring adverse events

Study monitoring and detection of any adverse events will take continuously throughout the study. There are minimal risks associated with this study and include fatigue and time involvement. All videos, prompts, and interview questions are not beyond what is ordinarily encountered in daily life or during the performance of routine psychological tests. Participants are given contact information and encouraged to reach out anytime to the study primary investigatory and/or the institution’s Office of Responsible Research Practices.

## Conclusion

This paper describes the protocol for the first study examining the feasibility, acceptability, and preliminary effects of the web-delivered parent training program, Attend Behavior. This study will be targeting a rural population that is not only underserved but also underrepresented in research. The data collected will provide rich insight into their needs when it comes to parent training programs. The intervention being studied is unique in that parents can access the intervention on their own, as well as clinicians who are not necessarily specialized to treat children with ASD can use the program with the families of their patients with ASD. With this in consideration, this intervention can not only reach rural families due to it being self-paced and web-delivered, but it also mitigates the issue of having a national shortage of clinicians specialized in ASD treatment.

This study will provide data that will enable us to identify critical areas for future program optimization and implementation. Intervention use data and subjective use experience interviews will allow us to identify areas for improvement and expansion. Through this pilot study approach, we will better understand whether a web-delivered parent support program will be effective and acceptable to rurally located families of children with ASD. Examining which recruitment routes were most valuable in increasing enrollment and what retention methods were reported to be effective by participants will help guide future larger studies to be more successful and efficient. As this study is aiming to recruit a hard-to-reach population that is understudied, findings about recruitment and retention efforts could not only influence future larger trials of Attend Behavior, but other intervention studies aiming to recruit rural parents of children with ASD as well. This study and the future trials that will be informed by the findings of this study will allow us to provide more evidence-based and personalized therapeutic tools to support families of children with ASD.

## Supporting information

S1 Checklist SPIRIT 2013 checklist: Recommended items to address in a clinical trial protocol and related items*. (DOC)

S1 File. S1 material Study protocol. (PDF)

S1 Table For list of acronyms used throughout the manuscript. (DOCX)
