# Supplementary material for: Feasibility, acceptability, and effects of a web-delivered behavioral parent training intervention for rural parents of children with autism spectrum disorder: A protocol
Source: PLoS One. 2024 Aug 27;19(8):e0307273. doi: 10.1371/journal.pone.0307273 (PMC11349109; doi:10.1371/journal.pone.0307273)
Supplement: S1 File. S1 material — (PDF) [file pone.0307273.s002.pdf]

Research Protocol

Evaluating a web delivered behavioral parent training intervention for rural parents of children with autism spectrum disorder

I. Objectives

The purpose of this study is to assess the acceptability, feasibility, and preliminary effects of a web-based parenting program, Attend Behavior, in a sample of parents/guardians of children with autism spectrum disorder (ASD) residing in rural areas.

The research questions that will be answered are:

1. Is Attend Behavior a feasible and acceptable intervention for rural parents/guardians of children with ASD?
2. How does Attend Behavior effect parental mental health (depression and stress) and child outcomes (disruptive behaviors and noncompliance in the home)?

II. Background and Rationale

Children with the neurodevelopmental condition Autism spectrum disorder (ASD) have distinct differences in language use, social interaction, and restricted or repetitive behavior, interests, or activities.[1] Some of the challenges that arise when parenting a child with ASD can leave parents feeling stressed and fatigued from disruptive behaviors, dependency level, and disrupted sleep cycles.[2–4] Parents of children with ASD have consistently reported higher levels of stress, depression, anxiety, social isolation, and poorer levels of physical health compared to parents of neurotypical children and parents of children with other various chronic health conditions (e.g. cerebral palsy, down syndrome).[2–13] The severity of ASD and disruptive behaviors have been correlated with higher parental stress.[3,14,15] Formal and informal emotional support sources have been shown to reduce stress for parents of children with ASD.[16–20] Traditional behavioral treatment approaches such as applied behavioral analysis (ABA), have been shown to benefit children with ASD;[21] however, approaches like ABA do not have high evidence for alleviating parent distress. Treatment approaches are delivered in various formats including parent-mediated delivery seen in parent training (PT) programs.[22] There is evidence to show that PT is effective in reducing child problem behaviors and parenting stress and depression.[23–27] In addition, there is economic benefits of PT interventions for children with behavioral issues as evidence by significant positive returns for investing in PT for youth at risk or already experiencing behavioral difficulties.[28]

Treatment disparities. Despite the evidence of effective ASD interventions, rural areas lack local support resources and specialized care for children with ASD.[29–32] These disparities include access to services, use of services, and satisfaction and perceived effectiveness of services for children with ASD.[31,33] The lack of providers in rural areas have resulted in many rural children with ASD relying on the school systems for interventions despite there being a lack of trained specialists and funding for special education programs in rural schools.[34,35] This is problematic as it results in children not receiving early intervention prior to entering the school system and parents being fearful for what will happen once they age out of school.[36] Intervention

delivery via telehealth services have been a response to the lack of service availability. Research evaluating the use of telehealth for those with ASD to provide behavioral therapies and PT programs has increased more recently in response to the COVID-19 pandemic. In a recent review of ASD services including assessments and intervention delivered via telehealth by Ellison et al.,[37] eleven studies were noted to be using telehealth platforms to deliver PT programs to parents of children with ASD, including one study examining the outcomes of the RUBI program delivered via telehealth.[22] Overall, results were very promising showing telehealth as an effective route of delivery for interventions.[37] It is important to note that in all eleven studies examining PT programs via telehealth in the review were using video conferencing platforms (e.g. Zoom) with a trained intervention provider being present.[37] Cantor et al.[29] noted that less than half of the mental health clinics in the US provided behavioral health care for children with ASD and only 12.7% reported having a clinician with specialized training for treating children with ASD. With this lack of trained ASD providers noted across the US, the use of telehealth services may expand the reach of services, but the reach will remain severely limited with the amount of ASD providers available.

Self-administered PT. In response to the treatment disparities noted and the lack of treatment providers nationally, self-administered PT programs may be key to closing the treatment gap. Such self-administered PT programs may be useful for parents to access independently as a primary intervention when a child is unable to be connected to a specialized provider, on a wait list for a diagnostic assessment or intervention services, or as an adjunct intervention. ***There remains a lack of research examining the feasibility, accessibility, and efficacy of self-delivered remote PT programs with rural parents.*** Parsons et al.[38] reviewed parent-mediated interventions delivered remotely for parents of children with ASD living outside urban areas and found seven studies examining interventions that included self-guided material (e.g. DVDs, websites, written material, videos). Some studies also included clinician interaction (remote coaching sessions, video conferencing). Findings from the review propose that remotely delivered PT interventions can improve child social behavior and communication skills and increase parental knowledge and skills.[38] However, the overall level of evidence was low due to various limitations of the studies (e.g. small samples, lack of standardized outcome measures) and only provides preliminary evidence.[38]

Evidence Supporting Attend Behavior. With the sharp increase in smartphone ownership in the last decade including nearly 80% of adults living in the rural US reporting owning a smartphone,[39] mobile-delivery of PT may be convenient for parents and offer more features than typical Web-based content (e.g. real-time behavior tracking). Attend Behavior is a mobile application that is available to download on the Apple App Store and Google Play. In addition, Attend Behavior is accessible on the Internet. Attend Behavior is available in both English and Spanish. Providing parents of children with ASD with a self-administered, mobile or web-based intervention may decrease parental stress by removing barriers of treatment wait times, transportation, childcare, time involvement, and geographical distance that parents of children with ASD living in rural areas face when participating in other in-person PT programs. The Attend Behavior PT intervention is customizable and aims to reduce a wide variety of child problem behaviors such as mild aggression, defiance, anger outbursts, impulsivity, and hyperactivity. Attend Behavior is also available in both English and Spanish. Attend Behavior content is based on the RUBI PT Program, henceforth referred to as RUBI. RUBI uses evidence-based parent-focused instruction with the basic principles of applied behavior analysis (ABA).

The goal of RUBI is to improve parent response to problem behaviors and prevent disruptive behaviors. RUBI has been shown to be feasible and have high acceptability from parents of children with ASD, improve parental knowledge, decrease parental stress, increase child's daily living skills, and decrease problem behaviors.[22–24,26,40] Historically, RUBI has been conducted in-person at a clinic with a provider and parent. Recently, with the COVID-19 pandemic, many providers had to pivot to use technology to provide services from distance. In response to this, Shanok et al.[26] completed a feasibility pilot study using synchronous telehealth services for delivering RUBI to parents of children with ASD. The findings of this study showed that the feasibility of RUBI delivered via telehealth for reducing problem behaviors of children with ASD was consistent with prior RUBI studies.[26] While Attend Behavior is used clinically and at times in addition to the RUBI program, there are no current studies published examining the effects of Attend Behavior for parents of children with ASD. There is an overall lack of published studies examining the effects of solely using a mobile-delivered PT program aimed at decreasing disruptive behaviors for children with ASD. One study noted using the application iSTIM to reduce stereotypy behavior.[41] Therefore, the proposed study will provide an important contribution to the major gap in knowledge surrounding mobile application delivered ASD PT.

Purpose

Therefore, the purpose of the proposed study is to test the acceptability, feasibility, and preliminary effects of Attend Behavior in an underserved population (rural) of parents of children with ASD. Attend Behavior was chosen for testing based on a rigorous review of the existing parenting programs conducted by the study investigator. Ultimately, Attend Behavior mirrors the evidence-based RUBI parent training program that shows promising results for reducing parental stress and evidence of effectiveness in reducing child behavior problems. Attend Behavior is available on the Web and as a mobile application and can be used independently by parents or in collaboration with a clinician, making it more accessible and versatile.

In this study, we will pilot test Attend Behavior in a sample of 40 parents/guardians with a young child (2-11 years old) with ASD. Parents will provide instrumental feedback on the feasibility and acceptability of Attend Behavior and provide feedback on additional elements that they need in an intervention to support them. Preliminary results of this pilot study will influence future larger randomized control trials.

III. Procedures

A. Research Design

The proposed pilot study is a single group pre/post-intervention design with both quantitative and qualitative data; therefore, a mixed methods design will be employed.

B. Sample

Parents will be recruited using the following methods:

1. Mid-Ohio Behavioral Health

The primary method of recruiting participants in this study is through a community mental health organization, Mid-Ohio Behavioral Health, which has multiple locations in central Ohio including Franklin, Licking, Muskingum, Guernsey, and Coshocton counties. Flyers to promote the study with a QR code and a printed link to direct interested clients to the Redcap survey for assessment of eligibility and collection of contact information will be posted in the Mid-Ohio Behavioral offices for study promotion. The study team will provide education regarding the study including study goals and general information via an email to all staff at Mid-Ohio Behavioral Health. The email will also include the link (to direct interested clients to the Redcap survey for assessment of eligibility and collection of contact information) to provide to their clients if they are interested in the study.

2. The Learning Spectrum School

A second recruitment site is The Learning Spectrum (TLS) School. TLS is a school focusing on providing education to children with ASD and other developmental disabilities. One of their sites located in Licking county and will be used for study promotion and recruitment. Flyers to promote the study with a QR code and a printed link to direct interested parents to the Redcap survey for assessment of eligibility and collection of contact information will be posted at TLS-Johnstown for study promotion. The study team will provide education regarding the study including study goals and general information via an email to all staff at TLS-Johnstown. The email will also include the link (to direct interested clients to the Redcap survey for assessment of eligibility and collection of contact information) to provide to their student's parents if they are interested in the study.

3. Facebook

A third method is Facebook. To recruit via Facebook, the study team will create a Facebook account containing relevant information about the research study's purpose, eligibility, and contact information to learn more. Using the research study's Facebook page, the research team will privately message Facebook group moderators of parents of children with ASD groups asking permission to post information about the study in private pages. With the permission of private group moderators, we will also post the study recruitment flyer in the private groups. This method of recruitment on Facebook has been previously used by the PI in studies as a research assistant and co-investigator and has been exceedingly successful. In addition, an ad on Facebook that was created with collaboration with the Ohio State University CCTS may be published for recruitment. The ad is available in both English and Spanish.

4. Agencies, schools, and public libraries

We will recruit from behavioral and neurodevelopmental clinics such as Nationwide Children's Hospital Center for Autism Spectrum Disorder and I Am Boundless. Schools in rural areas will be contacted as well such as Foxfire School in Muskingum county and My Place to Be in Licking county. Public schools in rural would be contacted to increase recruitment if needed such as Zanesville City Schools, West Muskingum Local Schools, Crooksville Exempted Village Schools, and New Lexington City School District.

Furthermore, rurally located public libraries and primary care offices will be contacted to engage in efforts of recruitment as well. We will send emails to representatives of the above-listed organizations (see template) asking them to pass along the study information and flyer to potentially interested parties. Alternatively, organizations can opt to print the study recruitment flyer and display the flyer in their facility if they so choose. In addition, postcards were created with collaboration with the Ohio States University CCTS and will be offered to be mailed to any organization that reports they would prefer the postcards.

5. The Ohio State University Extension Offices

Ohio State University (OSU) Extension offices located in rural counties such as Muskingum County, Holmes County, and Knox County will be contacted for aid in recruitment. We will send emails to representatives at the various OSU Extension Offices (see template) asking them to pass along the study information and flyer to potentially interested parties. Alternatively, organizations can opt to print the study recruitment flyer and display the flyer in their facility if they so choose.

6. Simons Foundation Autism Research Initiative (SFARI) Base

The SFARI Base has an online portal for the submission of research recruitment requests. Researchers can request access to data or submit a research match application to recruit individuals or families for future research studies. Cohorts include the Simons Simplex Collection, Simons Searchlight, Simons Foundation Powering Autism Research for Knowledge (SPARK), Autism Inpatient Collection, Autism BrainNet, and iPS Cell Models. A request for this project will be created in SFARI to be advertised for potential participants signed up in the SFARI Base to receive information regarding the study. Further information regarding the SFARI Base can be found here: https://www.sfari.org/resource/sfari-base.

Screening procedures

All interested potential participants will be directed by a link and/or QR code on the flyers to a website housing the Redcap survey to screen for eligibility. Participants will answer a short survey to determine eligibility. Only screening data from enrolled participants will be retained. All other screening data from unenrolled or ineligible participants will be destroyed immediately.

If the participant is eligible, they will be prompted to provide their name and contact information (i.e. phone number, preferred time of day for contact, email). The study PI or study team member will contact all eligible interested participants promptly. The verbal consent form will be read in full by the PI the participant over the phone. All consented participants will be emailed a copy of the consent form and contact card.

Inclusion criteria

a. The legal guardian of one or more children ages 2-11 years old with a formal diagnosis of ASD by parent report
b. Child with ASD resides at home with the parent
c. Parent age is 18 years or older

d. Reside in a rural (micropolitan or noncore) county
e. Has access to either a smartphone with the Apple App store or Google Play store or a computer with Internet access
f. Ability to read in English or Spanish

Exclusion

a. They are currently participating in another behavior PT program or in the past 6-months have been enrolled in another behavior PT program as this could confound the results

Sample Size

We will enroll up to 40 parents/guardians into the study. The sample size is deemed sufficient for a pilot study and as we are examining qualitatively feasibility and acceptability of Attend Behavior.

C. Measurement / Instrumentation

Data in this study will be both quantitative and qualitative in nature. Parents will complete self-reported surveys at baseline and post-intervention (T2) using REDCap. For baseline data collection, parents will be emailed or texted a link to the respective survey. There will be two baseline surveys, one for parents of children ages 2-5 years old and another for parents of children ages 6-11 years old. Baseline and T2 surveys will be available in both English and Spanish. For T2, participants will be emailed, texted, and/or messaged on Attend Behavior (participant preference) the link to the survey. The total time commitment for the baseline and T2 surveys is no longer than 30 minutes. Please see below for a description of study assessments:

1. The demographic survey will include both parent and child information that is self-reported by the parent. The demographic survey will be administered at baseline.
2. The Aberrant Behavior Checklist (ABC). The ABC measures child problem behavior and consists of 58 questions with five subscales: irritability, withdrawal, stereotypic behavior, hyperactivity, and inappropriate speech. The ABC will be administered at baseline and T2.
3. The Home Situations Questionnaire – Autism Spectrum Disorder (HSQ-ASD). The HSQ-ASD measures child noncompliance in the home and consists of a 24-item rating where parents report yes/no if the child has been noncompliant in the past 4 weeks to a variety of situations in the home setting (e.g. "when told to brush teeth"). If the child reports the child was noncompliant in a situation within the last 4 weeks, they are prompted to rate the severity of that problem on a 1-9 Likert scale with higher scores indicating higher severity. The HSQ-ASD will be administered at baseline and T2.
4. The PROMIS Depression – Short Form 6A. This 6-item PROMIS short form measurement prompts participants to rate how often they have felt emotions associated with depression within the past 7 days on a scale of 1 = *Never* to 5 = *Always*. The PROMIS will be administered at baseline and T2.
5. The Parenting Stress Index – Short Form (PSI-SF). The PSI-SF is a 36-item measurement to measure parental stress with three domains: parental distress, parent-child dysfunctional interaction, and difficult child to yield a total stress score. The PSI-SF will be administered at baseline and T2.

6. Acceptability of Intervention (AIM), Intervention Appropriateness Measure (IAM), and the Feasibility of Intervention Measure (FIM). With a total of 12-items answered on a Likert scale of 1 = *completely disagree* to 5 = *completely agree*, the AIM, IAM, and FIM is a brief measurement to examine implementation success.[42] The AIM, IAM, and FIM will be administered at T2.

7. The Autism Severity Rating Scale – Short Form (ASRS-SF) (2-5 year old and 6-18 year old). The ASRS-SF (2-5 year old) and the ASRS-SF (6-18 year old) are both 15-item measurement scales to assess the severity of common behaviors associated with Autism Spectrum Disorder. The ASRS-SF will only be given at the baseline survey. If the parent has a child that is 2-5 years old, they will be given the baseline survey that includes the ASRS-SF (2-5 year old) form. If the parent has a child that is 6-11 years old, they will be given the baseline survey that includes the ASRS-SF (6-18 year old) form.

Exit interviews

After the T2 survey is complete, participants will then complete a 20-minute exit interview with the PI. The exit interview will be conducted over the phone or zoom according to participant preference. The purpose of the post-intervention interview is to gauge the preliminary feasibility and acceptability of Attend Behavior. The interview will be guided by a semi-structured interview guide. Interviews will be audio-recorded and transcribed.

D. Detailed study procedures

Once participants have been screened and are eligible, the study PI or study team member will contact the participant for an enrollment meeting. The enrollment meeting will take approximately 15 minutes to complete and take place over the phone. The enrollment meeting will not be audio recorded. The enrollment meeting is comprised of the study team educating the participant on the study, obtaining consent, verifying participant contact information, and providing an overview of the Attend Behavior intervention. The participant will be texted or emailed a link to the baseline survey after the phone call is complete. Once the baseline survey is complete, the PI will email the participant the short video created by Attend Behavior explaining the features of Attend Behavior and how to use the program, a link to the Attend Behavior website, links to download the Attend Behavior mobile application in the Apple Store and the Google Play Store, and their access code to enable their Attend Behavior account.

The enrollment meeting will also include assessing the participant's preference for email, text messages, and/or Attend Behavior messaging reminders to complete the T2 survey and exit interview. Participants will be offered to schedule the exit interview during the enrollment appointment or an option to schedule the interview at a later time. Participants will be reminded that they can Zoom into the 1:1 interview or complete the interview via phone. Participants will be informed that they will receive a survey link after completing the intervention to complete approximately 12-weeks after initiating the intervention. They will be informed that the Attend

Behavior mobile application will be sending them reminders to complete learning modules weekly and to practice skills taught in the modules.

Attend Behavior

Attend Behavior consists of 12 learning modules with multiple mini lessons included in each module. Participants will be asked to complete one module per week for a total of 12 weeks. Participants are not compensated for completing the modules.

As previously stated, participants will complete assessments at baseline, T2, and an exit interview. Participants will be compensated a $25 e-gift card to Amazon (via email) after they complete the T2 survey. Participants who do not complete the T2 survey will not be compensated for this portion of the study.

Participants will be asked to complete a 1:1 semi-structured interview with the study PI or study team member at a date and time convenient to them via phone or Zoom (participant preference). The meeting will be audio recorded. The participant will be sent reminders three days prior and one day prior of their scheduled interview via email, text, and/or messaging on the Attend Behavior application. Participants who complete the exit interview will be emailed a $25 e-gift card to Amazon for their participation. Participants who do not complete the exit interview will not be compensated for this portion of the study.

Potential Risks

All of the research related procedures, surveys, and questionnaires are to be reviewed by the IRB under §45 CFR 46.404, unless deemed otherwise by the IRB. Study staff are committed to minimizing risks even when risks are minimal. Potential risks related to the proposed study include coercion, the actual or perceived loss of confidentiality, participant burden, and a risk related to data collection in that participants may feel uncomfortable being recorded or answering some of the questions asked. There are no known physical risks associated with participating in the proposed study. There are no alternative treatments or procedures.

Protection Against Risks

Potential risks will be minimized by strictly adhering to the guidelines for research outlined by the IRB, state law, HIPAA, and the DHHS Federal Policy for the Protection of Human Subjects (45 CFR Part 46 Subpart D). There will be no costs to participate in the study. Coercion: Consent forms will state that participation in the project will not impact their ability or their child's ability to access services or receive care. Participant Burden: Participants will be assured that they are free to withdraw from the research at any time. The study team was sensitive to participant burden in selecting the meeting times and measures.

Confidentiality: Audio Recordings

Participants will be informed at enrollment and again prior to beginning the study that portions of their participation may be audio recorded. Specifically, exit interviews will be audio-recorded in Zoom. Zoom is a secure, user-friendly, cloud-based enterprise videoconferencing service that Ohio State University implemented in 2018. The meeting host controls all meeting features,

which include mute/unmute participants, screen sharing, recording options, video sharing, remote screen control and participant annotation. Audio recordings will be de-identified by transcribing the data and removing any names or titles that could link the comments with an identifiable person. Zoom is accessed via Ohio State's single sign-on solution, which provides an environment in which users can authenticate/log in at one time to a central server and connect with web-based services. Meeting security best practices such as waiting lobby, passwords to enter rooms, inability to join before host, disabling of sharing for participants, and disabling annotation by participants by default have been implemented. Participants will be informed that they can decline to answer any questions and may discontinue participation at any point.

Confidentiality: Data Collection.

All data will be collected in REDCap in which participants will be texted or emailed a link to the respective survey. Encryption used by REDCap to protect respondents includes hashed passwords and SSL certificates between client and server using the secure HTTPS protocol. REDCap allows for personalized hashed study ID links for each parent. REDCap offers a Data Export Utility, which enables researchers to export their data in an automated manner into formats that are compatible with commonly used statistical analysis packages, such as SAS, SPSS, Stata, and R. All protected health information can be flagged and excluded on export to statistical analysis software. Each REDCap project has function to delete collected data in a single request.

Confidentiality: Data Storage.

To protect confidentiality, all study records will be coded by identification numbers and the key linking names with numbers will be kept in a separate location under the control of the study team. This list will be destroyed by the completion of the project.

There will be no hard copies of data in this study. All data (e.g., audio recordings, surveys) will be housed in the Ohio State University College of Nursing's R-drive. Only research personnel (i.e., RA, study investigators) will have access to the R-drive. The R-drive runs Windows Server 2012R2 and is only used to host research files. The Windows operating system is kept up to date by weekly, automatic updates from Microsoft. The physical server is housed in a secure room within Newton Hall. Only pertinent IT staff and the building coordinator have keys to access this room. Electronic access to the research server is restricted to the college LAN or VPN by a Cisco ASA 5510 firewall. Access from the internet without VPN is not allowed. Electronic access is further restricted by the on-host Windows Firewall. User access is tightly controlled, by request only. New users must be cleared by the College of Nursing Associate Dean for Research. Permissions to individual folders and subfolders are managed individually. Users only have access to folders of projects in which they are directly involved. All changes are submitted in writing. Permissions are audited annually by IT in collaboration with the folder/study owner. The administrator account for the server has a non-standard password and has been disabled. All user accounts connecting to the server are domain accounts and are authenticated through a domain controller. Accounts are automatically locked out for 30 minutes after 5 invalid attempts. User access and system information is logged and exported to a central event logging server. All data will be retained for seven years and then destroyed per the policy at Ohio State University.

Data will only be presented in aggregate with no disclosure of individual cases at scientific conferences or in publications. Data will only be transferred to others for analysis when the data is completely de-identified. Five years after the last follow up assessment is completed; any identifying information will be destroyed. Per the American Psychological Association guidelines, the de-identified data will be kept seven years after the final publication of these data.

Discomfort with Questions. We do not anticipate discomfort with questions across study phases as we are asking participants to provide demographic information and to evaluate an intervention. Adverse reactions from answering questions in the assessments are unlikely because these are standard questionnaires used across many populations without complication. Any distress will be minimized by assurances that participants can skip questions they do not feel comfortable answering and withdraw from the study at any time without penalty. Participants will be encouraged that if at any time during the study they feel distressed, they are encouraged to contact the study team.

Specific Procedures for Abuse, Homicide, and Suicide.
All consent forms will include a statement that if a member of the research team suspects that a child is being abused or neglected, they are required by law to report the concern to child protective services. If the parent has any concern about this, the consent document will encourage the parent to call the toll-free number to discuss those concerns before signing the consent form. Ohio State law mandates that workers in certain professions (e.g., RNs) report if they have reasonable cause to suspect abuse or neglect. Although we do not ask about child abuse or neglect, if during the course of the study something triggers the concern of anyone on the research team related to child abuse or neglect, we will institute procedures for mandated reporting. Public Children's Services Agency (PCSA) of the State of Ohio will be notified by phone and may initiate an investigation. In addition to the placement of the phone call, research staff will send written confirmation to the appropriate child protective services field office within 48 hours. These procedures will satisfy all legal requirements. We do not ask about homicidal or suicidal intent or ideation in this study. However, we have protocols in place should a parent reveal homicidal or suicidal intent or ideation. In the case of homicidal intent, we will report the information to both the police and the intended victim. In the case of suicidal ideation or intent, the PI will assess for a plan, means, and intent. Upon this review, next steps are discussed. For parents who are not deemed acutely suicidal, we will provide a list of referrals for mental health services within the area. If imminent risk is determined in either a parent or child, the participant will be directed to go to the emergency room. If the participant refuses to go to the emergency room and imminent risk of suicide/harm to self is determined, then we will call the police to accompany the participant. If the child's disruptive behavior becomes severe and places the child or others around them at possible harm, the parent will be given a list of resources for higher level of care within the area.

E. Internal Validity

As this is pilot study with a small sample size, there are inherent risks to validity. This study

includes a small sample size and therefore, the findings are not generalizability to the broader population of parents with children with ASD. Multiple validated questionnaires are being used to assess outcome data for preliminary effects to reduce threats to internal validity.

F. Data Analysis

Quantitative Data

Quantitative data in this study is derived from the demographic questionnaire, baseline survey, and the post-intervention survey. We will conduct exploratory data analyses to check data accuracy, examine variable distributions, and summarize sample characteristics. Data anomalies once identified will be fully investigated and remedial strategies will be considered as appropriate. Although the full ABC measurement will be included in the baseline and T2 surveys, only the ABC-Irritability subscale (ABC-I) will be used for analyses as the intervention does not target the other symptoms included in the other ABC subscales. For Aim 1, we will use descriptive statistics to examine the acceptability and feasibility of the intervention, including rates of completion of intervention and recruitment and retention rates, as well as average scores of AIM, IAM, and FIM. For Aim 2, descriptive statistics will be used to summarize each outcome (i.e. ABC-I, HSQ-ASD, PROMIS, and PSI-SF). We will report mean differences, 95% confidence intervals, and effect sizes for baseline to post-intervention difference. Mixed-effects models with subject-specific random effects will be further applied to test the intervention effects adjusted for covariates if any. The covariates will be included as fixed-effects in the model and potential interaction effects will be examined. This approach will allow us to account for missing data under the missing at random assumption.

Qualitative data

Qualitative data sources in this study include the exit interview. All audio files will be transcribed verbatim promptly after the interviews. The original audio recordings and transcripts will be stored in the College of Nursing R-drive. A member of the study team will review the transcripts of the audio interviews for accuracy. Prior to data analysis, all qualitative sources will be de-identified with identifiers being redacted. All qualitative data will be analyzed using NVIVO.

NVIVO will assist with the organization and coding of the unstructured narrative data. NVIVO has several capabilities including classifying and arranging data and organizing the data into similar concepts to allows for relationships to emerge. The PI and a member of the research team will read all participant interviews. Codes and coding categories will be compared and discrepancies discussed before establishing a codebook.

Due to the qualitative methodology chosen, data analysis will occur simultaneously with data collection. Data saturation will be attained when no new additional data are revealed in the conceptual categories. The data analysis strategy that will be used in the proposed study is content analysis due to the inductive nature of the work. The aim of content analysis is to provide a thick, rich description of the phenomena of interest using the participant's own words. In qualitative description, the researcher does not infer but rather stays close to the data and will

often use quotes, modifiable codes, and frequency of codes. To ensure credibility of the data, the PI will keep a "reflective commentary" log. The purpose of the reflective commentary log is to acknowledge biases and record initial impressions as well as patterns as they emerge in the data.

IV. Bibliography

1. American Psychiatric Association, American Psychiatric Association, eds. *Diagnostic and Statistical Manual of Mental Disorders: DSM-5*. 5th ed. American Psychiatric Association; 2013.

2. Almansour MA, Alateeq MA, Alzahrani MK, Algeffari MA, Alhomaidan HT. Depression and anxiety among parents and caregivers of autistic spectral disorder children. *Neurosciences (Riyadh)*. 2013;18(1):58-63.

3. Benson PR. The Impact of Child Symptom Severity on Depressed Mood Among Parents of Children with ASD: The Mediating Role of Stress Proliferation. *Journal of Autism and Developmental Disorders*. 2006;36(5):685-695. doi:10.1007/s10803-006-0112-3

4. Catalano D, Holloway L, Mpofu E. Mental Health Interventions for Parent Carers of Children with Autistic Spectrum Disorder: Practice Guidelines from a Critical Interpretive Synthesis (CIS) Systematic Review. *International Journal of Environmental Research and Public Health*. 2018;15(2):341. doi:10.3390/ijerph15020341

5. Antezana L, Scarpa A, Valdespino A, Albright J, Richey JA. Rural Trends in Diagnosis and Services for Autism Spectrum Disorder. *Frontiers in Psychology*. 2017;8. doi:10.3389/fpsyg.2017.00590

6. Al-Oran HM, AL-Sagarat AY. Parenting Stress of Children with Autistic Disorder. *OALib*. 2016;03(07):1-10. doi:10.4236/oalib.1102791

7. Feinberg E, Augustyn M, Fitzgerald E, et al. Improving Maternal Mental Health After a Child's Diagnosis of Autism Spectrum Disorder: Results From a Randomized Clinical Trial. *JAMA Pediatrics*. 2014;168(1):40. doi:10.1001/jamapediatrics.2013.3445

8. Hayes SA, Watson SL. The Impact of Parenting Stress: A Meta-analysis of Studies Comparing the Experience of Parenting Stress in Parents of Children With and Without Autism Spectrum Disorder. *J Autism Dev Disord*. 2013;43(3):629-642. doi:10.1007/s10803-012-1604-y

9. Neely-Barnes SL, Dia DA. Families of children with disabilities: A review of literature and recommendations for interventions. *Journal of Early and Intensive Behavior Intervention*. 2008;5(3):93-107. doi:10.1037/h0100425

10. Padden C, James JE. Stress among Parents of Children with and without Autism Spectrum Disorder: A Comparison Involving Physiological Indicators and Parent Self-Reports.

Journal of Developmental and Physical Disabilities. 2017;29(4):567-586. doi:10.1007/s10882-017-9547-z

11. Yorke I, White P, Weston A, Rafla M, Charman T, Simonoff E. The Association Between Emotional and Behavioral Problems in Children with Autism Spectrum Disorder and Psychological Distress in Their Parents: A Systematic Review and Meta-analysis. *J Autism Dev Disord*. 2018;48(10):3393-3415. doi:10.1007/s10803-018-3605-y

12. Lovell B, Moss M, Wetherell MA. The psychophysiological and health corollaries of child problem behaviours in caregivers of children with autism and ADHD: Problem behaviours in autism and ADHD caregivers. *J Intellect Disabil Res*. 2015;59(2):150-157. doi:10.1111/jir.12081

13. Merkaj V, Kika M, Simaku A. Symptoms of Stress, Depression and Anxiety between Parents of Autistic Children and Parents of Tipically Developing Children. *Academic Journal of Interdisciplinary Studies*. Published online July 1, 2013. doi:10.5901/ajis.2013.v2n2p345

14. Miranda A, Mira A, Berenguer C, Rosello B, Baixauli I. Parenting Stress in Mothers of Children With Autism Without Intellectual Disability. Mediation of Behavioral Problems and Coping Strategies. *Front Psychol*. 2019;10:464. doi:10.3389/fpsyg.2019.00464

15. Tomeny TS. Parenting stress as an indirect pathway to mental health concerns among mothers of children with autism spectrum disorder. *Autism*. 2017;21(7):907-911. doi:10.1177/1362361316655322

16. Ault S, Breitenstein SM, Tucker S, Havercamp SM, Ford JL. Caregivers of children with autism spectrum disorder in rural areas: A literature review of mental health and social support. *Journal of Pediatric Nursing*. 2021;61:229-239. doi:10.1016/j.pedn.2021.06.009

17. Benson PR. Network Characteristics, Perceived Social Support, and Psychological Adjustment in Mothers of Children with Autism Spectrum Disorder. *J Autism Dev Disord*. 2012;42(12):2597-2610. doi:10.1007/s10803-012-1517-9

18. Ekas NV, Lickenbrock DM, Whitman TL. Optimism, Social Support, and Well-Being in Mothers of Children with Autism Spectrum Disorder. *J Autism Dev Disord*. 2010;40(10):1274-1284. doi:10.1007/s10803-010-0986-y

19. Kuru N, Piyal B. Perceived social support and quality of life of parents of children with Autism. *Niger J Clin Pract*. 2018;21(9):1182-1189. doi:10.4103/njcp.njcp_13_18

20. Lovell B, Moss M, Wetherell MA. With a little help from my friends: Psychological, endocrine and health corollaries of social support in parental caregivers of children with autism or ADHD. *Research in Developmental Disabilities*. 2012;33(2):682-687. doi:10.1016/j.ridd.2011.11.014

21. Chahin SS, Apple RW, Kuo KH, Dickson CA. Autism spectrum disorder: psychological and functional assessment, and behavioral treatment approaches. *Transl Pediatr*. 2020;9(S1):S66-S75. doi:10.21037/tp.2019.11.06

22. Bearss K, Burrell TL, Challa SA, et al. Feasibility of Parent Training via Telehealth for Children with Autism Spectrum Disorder and Disruptive Behavior: A Demonstration Pilot. *J Autism Dev Disord*. 2018;48(4):1020-1030. doi:10.1007/s10803-017-3363-2

23. Bearss K, Johnson C, Smith T, et al. Effect of Parent Training vs Parent Education on Behavioral Problems in Children With Autism Spectrum Disorder: A Randomized Clinical Trial. *JAMA*. 2015;313(15):1524. doi:10.1001/jama.2015.3150

24. Edwards GS, Zlomke KR, Greathouse AD. RUBI parent training as a group intervention for children with autism: A community pilot study. *Research in Autism Spectrum Disorders*. 2019;66:101409. doi:10.1016/j.rasd.2019.101409

25. Iadarola S, Levato L, Harrison B, et al. Teaching Parents Behavioral Strategies for Autism Spectrum Disorder (ASD): Effects on Stress, Strain, and Competence. *J Autism Dev Disord*. 2018;48(4):1031-1040. doi:10.1007/s10803-017-3339-2

26. Shanok NA, Lozott EB, Sotelo M, Bearss K. Community-based parent-training for disruptive behaviors in children with ASD using synchronous telehealth services: A pilot study. *Research in Autism Spectrum Disorders*. 2021;88:101861. doi:10.1016/j.rasd.2021.101861

27. Iida N, Wada Y, Yamashita T, Aoyama M, Hirai K, Narumoto J. Effectiveness of parent training in improving stress-coping capability, anxiety, and depression in mothers raising children with autism spectrum disorder. *Neuropsychiatric Disease and Treatment*. 2018;Volume 14:3355-3362. doi:10.2147/NDT.S188387

28. McDaid D, Park AL, Wahlbeck K. The Economic Case for the Prevention of Mental Illness. *Annu Rev Public Health*. 2019;40(1):373-389. doi:10.1146/annurev-publhealth-040617-013629

29. Cantor J, McBain RK, Kofner A, Stein BD, Yu H. Fewer Than Half Of US Mental Health Treatment Facilities Provide Services For Children With Autism Spectrum Disorder: Results from a survey of US mental health treatment facilities on the availability of behavioral health care services for children with autism spectrum disorder. *Health Affairs*. 2020;39(6):968-974. doi:10.1377/hlthaff.2019.01557

30. Elder JH, Brasher S, Alexander B. Identifying the Barriers to Early Diagnosis and Treatment in Underserved Individuals with Autism Spectrum Disorders (ASD) and Their Families: A Qualitative Study. *Issues in Mental Health Nursing*. 2016;37(6):412-420. doi:10.3109/01612840.2016.1153174

31. Maria P. Mello, Samantha E. Goldman, Richard C. Urbano, Robert M. Hodapp. Services for children with autism spectrum disorder: Comparing rural and non-rural communities. *Education and training in autism and developmental disabilities*. 2016;51(4):355-365.

32. McBain RK, Kareddy V, Cantor JH, Stein BD, Yu H. Systematic Review: United States Workforce for Autism-Related Child Healthcare Services. *Journal of the American Academy of Child & Adolescent Psychiatry*. 2020;59(1):113-139. doi:10.1016/j.jaac.2019.04.027

33. Murphy MA, Ruble LA. A Comparative Study of Rurality and Urbanicity on Access to and Satisfaction with Services for Children with Autism Spectrum Disorders. *Rural Special Education Quarterly*. 2012;31(3):3-11. doi:10.1177/875687051203100302

34. Antezana L, Scarpa A, Valdespino A, Albright J, Richey JA. Rural Trends in Diagnosis and Services for Autism Spectrum Disorder. *Frontiers in Psychology*. 2017;8. doi:10.3389/fpsyg.2017.00590

35. Pennington R, Horn C, Berrong A. An Evaluation of the Differences between Big City and Small Town Special Education Services for Students with Low Incidence Disabilities in Kentucky. *Rural Special Education Quarterly*. 2009;28(4):3-9. doi:10.1177/875687050902800402

36. Walker A, Alfonso ML, Colquitt G, Weeks K, Telfair J. "When everything changes:" Parent perspectives on the challenges of accessing care for a child with a disability. *Disability and Health Journal*. 2016;9(1):157-161. doi:10.1016/j.dhjo.2015.06.002

37. Ellison KS, Guidry J, Picou P, Adenuga P, Davis TE. Telehealth and Autism Prior to and in the Age of COVID-19: A Systematic and Critical Review of the Last Decade. *Clin Child Fam Psychol Rev*. 2021;24(3):599-630. doi:10.1007/s10567-021-00358-0

38. Parsons D, Cordier R, Vaz S, Lee HC. Parent-Mediated Intervention Training Delivered Remotely for Children With Autism Spectrum Disorder Living Outside of Urban Areas: Systematic Review. *J Med Internet Res*. 2017;19(8):e198. doi:10.2196/jmir.6651

39. Martin M. Computer and Internet Use in the United States: 2018. Published 2021. https://www.census.gov/content/dam/Census/library/publications/2021/acs/acs-49.pdf

40. Scahill L, Bearss K, Lecavalier L, et al. Effect of Parent Training on Adaptive Behavior in Children With Autism Spectrum Disorder and Disruptive Behavior: Results of a Randomized Trial. *Journal of the American Academy of Child & Adolescent Psychiatry*. 2016;55(7):602-609.e3. doi:10.1016/j.jaac.2016.05.001

41. Trudel L, Lanovaz MJ, Préfontaine I. Brief Report: Mobile Technology to Support Parents in Reducing Stereotypy. *J Autism Dev Disord*. 2021;51(7):2550-2558. doi:10.1007/s10803-020-04735-6

42. Proctor E, Silmere H, Raghavan R, et al. Outcomes for Implementation Research: Conceptual Distinctions, Measurement Challenges, and Research Agenda. *Adm Policy Ment Health*. 2011;38(2):65-76. doi:10.1007/s10488-010-0319-7
