# Supplementary material for: Feasibility, acceptability, and effects of a web-delivered behavioral parent training intervention for rural parents of children with autism spectrum disorder: A protocol
Source: PLoS One. 2024 Aug 27;19(8):e0307273. doi: 10.1371/journal.pone.0307273 (PMC11349109; doi:10.1371/journal.pone.0307273)
Supplement: S1 Table — (DOCX) [file pone.0307273.s003.docx]

**Table 2. Acronym key.**

| Autism Spectrum Disorder | ASD |
| --- | --- |
| Parent Training | PT |
| Applied Behavioral Analysis | ABA |
| Research Units in Behavioral Intervention | RUBI |
| United States | U.S. |
| Home Situations Questionnaire – Autism Spectrum Disorder | HSQ-ASD |
| Autism Spectrum Rating Scale – Short Form | ASRS-SF |
| Aberrant Behavior Checklist | ABC |
| Aberrant Behavior Checklist – Irritability | ABC-I |
| Parenting Stress Index – Short Form | PSI-SF |
| Parental Distress | PD |
| Parent-Child Dysfunctional Interaction | PCDI |
| Difficult Child | DC |
| Acceptability of Intervention | AIM |
| Intervention Appropriateness Measure | IAM |
| Intervention Appropriateness Measure | FIM |
